# Socioeconomic and geographic variations in antenatal care coverage in Angola: further analysis of the 2015 demographic and health survey

**DOI:** 10.1186/s12889-020-09320-1

**Published:** 2020-08-15

**Authors:** Gebretsadik Shibre, Betregiorgis Zegeye, Dina Idriss-Wheeler, Bright Opoku Ahinkorah, Olanrewaju Oladimeji, Sanni Yaya

**Affiliations:** 1 Department of Reproductive, Family and Population Health, School of Public Health, Addis Ababa University, Addis Ababa, Ethiopia; 2 Shewarobit Field Office, HaSET Maternal and Child Health Research Program, Addis Ababa, Ethiopia; 3 Interdisciplinary School of Health Sciences, University of Ottawa, Ottawa, Canada; 4 The Australian Centre for Public and Population Health Research, Faculty of Health, University of Technology Sydney, Ultimo, NSW Australia; 5 Department of Public Health, Walter Sisulu University, Mthatha, Eastern Cape South Africa; 6 Faculty of Health Sciences, Durban University of Technology, Durban, South Africa; 7 Department of Family Medicine and Public Health, Faculty of Medicine, University of Botswana, Gaborone, Botswana; 8 School of International Development and Global Studies, Faculty of Social Sciences, University of Ottawa, 120 University Private, Ottawa, ON K1N 6N5 Canada; 9 The George Institute for Global Health, The University of Oxford, Oxford, UK

**Keywords:** Antenatal care, Inequality, Health disparities, Global health; Angola, DHS

## Abstract

**Background:**

In African countries, including Angola, antenatal care (ANC) coverage is suboptimal and maternal mortality is still high due to pregnancy and childbirth-related complications. There is evidence of disparities in the uptake of ANC services, however, little is known about both the socio-economic and geographic-based disparity in the use of ANC services in Angola. The aim of this study was to assess the extent of socio-economic, urban-rural and subnational inequality in ANC coverage in Angola.

**Methods:**

We analyzed data from the 2015 Angola Demographic and Health Survey (ADHS) using the World Health Organization (WHO) Health Equity Assessment Toolkit (HEAT) software. The analysis consisted of disaggregated ANC coverage rates using four equity stratifiers (economic status, education, residence, and region) and four summary measures (Difference, Population Attributable Risk, Ratio and Population Attributable Fraction). To measure statistical significance, an uncertainty interval (UI) of 95% was constructed around point estimates.

**Results:**

The study showed both absolute and relative inequalities in coverage of ANC services in Angola. More specifically, inequality favored women who were rich (D = 54.2, 95% UI; 49.59, 58.70, PAF = 43.5, 95% UI; 40.12, 46.92), educated (PAR = 19.9, 95% UI; 18.14, 21.64, *R* = 2.14, 95% UI; 1.96, 2.32), living in regions such as Luanda (D = 51.7, 95% UI; 43.56, 59.85, *R* = 2.64, 95% UI; 2.01, 3.26) and residing in urban dwellings (PAF = 20, 95% UI; 17.70, 22.38, PAR = 12.3, 95% UI; 10.88, 13.75).

**Conclusion:**

The uptake of ANC services were lower among poor, uneducated, and rural residents as well as women from the Cuanza Sul region. Government policy makers must consider vulnerable subpopulations when designing needed interventions to improve ANC coverage in Angola to achieve the 2030 Sustainable Development Goal of reducing global maternal mortality ratio to 70 deaths per 100,000 live births.

## Background

Although maternal mortality declined from 451,000 in 2000 to 295,000 in 2017, more than 800 women still die globally each day from pregnancy and childbirth-related complications [1]. Approximately 20 other women undergo disabilities, severe injuries or infections for each maternal death. Globally, sub-Saharan Africa accounts for over two thirds (68%) of all maternal deaths every year, which is roughly 533 maternal deaths per 100,000 live births, or 200,000 maternal deaths a year [1]. Although there was a drop of 58.9 deaths per 100, 000 live births in the maternal mortality ratio (MMR) in Angola between 1990 and 2015, the MMR was 477 deaths per 100,000 live births at the end of the 2015 Millennium Development Goals [2].

Antenatal care (ANC) is the health care provided by a skilled professional during pregnancy [3]. ANC coverage is a measure of access and utilization of health care during pregnancy [4]. It provides an opportunity for pregnant women to use services that contribute to a “positive pregnancy experience” [3]. ANC coverage remains an essential indicator of access and use of health care during pregnancy; it is measured as the number of women aged 15–49 with a live birth in a given period that received ANC, four or more times during pregnancy, as a percentage of the women aged 15–49 who had a live birth in the same period [5]. Numerous studies have shown positive effects of antenatal care on infant birth weight [6, 7], early detection of diseases or risks and fetal abnormalities including the diagnosis of growth retardation [8–10] and reductions in maternal and neonatal morbidity and mortality [11, 12].

Studies conducted by a number of scholars in Africa have identified socio-economic factors such as maternal education, household wealth status, sub-national region, and place of residence as predictors of the utilization of ANC services [13–19]. Literature in Angola show maternal health service, including ANC, can be affected by socio-economic factors such as maternal education, maternal age, household economic status, place of residence, distance to facility, parity and previous adverse pregnancy outcomes [20, 21].

Monitoring the status of inequality, both across and within countries, identifies the prevalence of inequality in various facets of health and considers priority areas for further research. Policies, programmes and practices should ensure equity by improving the lives of the most disadvantaged subgroup(s). Without a dedicated focus of the investments, initiatives may lead to an increase in national coverage but the risk remains in intensifying inequality within the country as inequities among the sub-groups are not addressed [22]. Maternal health disparities have been key issues across [23] and within countries [24, 25]. A study in low-and middle-income countries (LMICs) shows ANC coverage of at least four visits differed by 27 percentage points between women who attended secondary school and above and non-educated women [26]. Furthermore, ANC coverage of at least one visit different by nearly 11 percentage points between richest and poorest women in half of the studied countries [26].

There is little evidence in Angola about determinants of utilization of ANC services [20, 21] and the extent of socio-economic and geographically related inequalities are largely unknown. Context-based evidence is necessary to provide targeted interventions that reduce health inequities among sub-groups [27]. Therefore, this study determined the magnitude of socio-economic, urban-rural and regional disparities in Angola using the rigorous inequality measuring techniques, the Health Equity Assessment Toolkit, developed by the WHO. Findings from the study will contribute to literature on inequalities in maternal healthcare services utilization in low-and middle-income countries and toward the attainment of universal health coverage as defined in the Sustainable Development Goals.

## Methods

### Study area

Angola is a vast country with a total population of more than 30.8 million (2018) [28]. Despite substantial progress on macro-economic constancy and fundamental improvements, Angola is still downgraded by the effects of decreased oil fees and an estimated reduction in gross domestic product (GDP) of about 1.2% in 2018 [28]. Oil accounts for one-third of the GDP and more than 90% of exports in the country [28]. Angola is ranked number 147 out of 189 countries in the Human Development Index despite its vast oil wealth and high per capita GDP. This implies medium scores in the education and health sectors of the economy and scores above the average of 0.541 for countries in Sub-Saharan Africa [29]. Much of the population is living in poverty, and the country could benefit from more inclusive development policies as the government establishes a social protection system program intended to support deprived societies [28].

Angola’s health system comprises of two major divisions, primary health with a community-level preventative service, and acute care with hospital services for complex treatments. According to the 2017 UNICEF report, the country has also been affected by various disasters such as cholera outbreaks, floods and a refugee inflow from the Democratic Republic of the Congo, while continuing to fight against the effects of the El Niño-induced droughts and the yellow fever outbreak [30].

### Data source

We used data from the 2015-2016 Angola Nationally representative Multiple Indicator and Health Survey (IIMS). This is the fourth Multiple Indicator Cluster Survey and the first Demographic and Health Survey for the country. It aimed to provide a profile of the country’s demographic and health situation regarding maternal and child health, fertility, family planning status, malaria, HIV/AIDS, and domestic violence [21]. A total of 14,379 women aged 15–49 in 16,109 households and 5684 men aged 15–54 in half of the selected households were interviewed in the 2015–2016 IIMS. This represented a response rate of 96% for women and 94% for men. The sample design for the 2015–16 IIMS provided national and regional as well as rural-urban estimates [21]. Detailed information on study design (i.e. sample weights to account for stratified survey design used by the DHS) are available in the survey and the details of how the WHO accounted for this in the HEAT Software can be found in the HEAT technical document [31].

### Selection of variables

ANC includes the number of antenatal care visits completed by a pregnant woman. In this study, inequality was measured for ANC coverage, where the woman received antenatal care at least four times during her last pregnancy. Although WHO has recently made new recommendations that the minimum number of antenatal visits should be eight [3], we used the previous WHO ANC visit recommendation and coverage definition of at least four ANC visits as a benchmark for a pregnant woman to be deemed protected from pregnancy-related risk and complications [32]. This article will refer to the 'at least four visit model' as ANC from hereon in.

### Measures

We measured inequality of ANC coverage using four equality stratifiers namely, economic status, educational status, place of residence and subnational region. Economic status was approximated by wealth index computed based on household assets and characteristics of the household. In DHS, wealth index is computed using Principal Component Analysis (PCA) [33]. It is classified as poorest, poor, middle, rich and richest. Educational status of the mother was categorized as no education, primary and secondary or higher education. Residence was classified as urban and rural. Subnational region was grouped into 18 regions for which the data was collected.

### Statistical analysis

Inequality in ANC coverage was disaggregated by four equity stratifiers (economic status, education status, place of residence and subnational region). Disparity was examined using the inequality summary measures of Difference, Population Attributable Risk (PAR), Population Attributable Fraction (PAF) and Ratio. While Difference and Ratio are simple measures, the remaining two are complex measures. Relative inequalities were measured by Ratio and PAF, whereas, the absolute inequalities were by Difference and PAR. By considering previous recommendation of using relative and absolute as well as single and complex measures in a single study, our selection of summary measures complied with and used all recommendations [22]. The significance of this approach is that each inequality summary measure could lead to different, even contradictory conclusions [22], and reduce bias in the decision-making process. Unlike simple measures, they consider the size of categories of a sub-population and are preferred when a population shift has possibly occurred [22]. Simple measures are easy for explanation, interpretation and understanding. Hence, an inequality study should comprise both simple and complex, as well as relative and absolute measures to provide a more comprehensive view for decision-makers.

The 2019 updated version of the 3.1 WHO’s Health Equity Assessment Toolkit (HEAT) software was used for the analysis [31]. Detailed procedures and calculation of summary measures are available in the HEAT software (31, and in the WHO handbook on health inequality monitoring [22]. But briefly, Difference (D) was calculated for economic status (richest group minus poorest group), education (secondary or higher educated group minus uneducated group), place of residence (urban minus rural) and subnational region (the region with highest ANC 4 coverage minus region with lowest ANC 4 coverage). Ratio (R) was calculated by dividing the two subgroups mentioned for each dimension to render a relative value. Inequality did not exist if D had a value of 0 or R had a value of 1.

PAR was also calculated by subtracting ANC coverage of the national average from the reference group. The reference group for economic status, education status and place of residence were richest, secondary school or higher and urban residence, respectively. For the subnational region, the reference group was the region with highest ANC estimate (Luanda). PAF is calculated by dividing PAR by the national average (μ) and multiplying by 100. A PAR or PAF value of zero indicated the absence of inequality and the greater absolute value of both complex measures indicated higher inequality favouring the advantaged subgroups. To examine whether ANC service demonstrates statistically significant inequalities across the sub-populations of each equity stratifier, we computed 95% Uncertainty Intervals (UI) around point estimates of each measure. For inequality measures of D, PAF and PAR, the lower and upper bounds of the UI must not include zero to interpret that inequality exists. For Ratio, the interval should not include one.

### Ethical consideration

From publicly available DHS data set, data analysis were performed. Because the ethical clearance was approved by the institution that commissioned, funded and managed the overall DHS program, further ethical clearance was not required. Informed consent from the participants before survey was ensured by those responsible for survey deployment. The ICF International and respective country’s ethical review board (IRB) also ensured that the protocols follow the U.S. Department of Health and Human Services regulations for the protection of human subjects.

## Results

In this study, a total of 8492 study participants were surveyed. Approximately 3046 (35.8%) were rural residents, more than one-fourth (26.8%) were non-educated, 3219 (37.9%) had primary school education and 2995 (35.2%) had attended a secondary school or higher. Regarding economic status, nearly one-fifth (19.7%) of the participants were from wealth quintile one (poorest) whereas, 1869 (22%), 1707(20.1%) and 1422 (16.7%) belonged to poorer, rich and richest subpopulations, respectively.

Table 1 shows the difference in ANC coverage across socio-economic and regional subpopulations in 2015 in Angola, with lower coverage among disadvantaged subgroups such as poorest, non-educated, rural residents, and those living in regions like Cuanza Sul.

**Table 1:** Coverage of ANC across subnational region, socio-economic and urban-rural subpopulations: Evidence from 2015 Angola demographic and health survey

Subgroup	Estimate (95% UI)	Population
**Economic status**		
Quintile 1 (poorest)	34.03 (30.44, 37.82)	1674
Quintile 2	44.82 (41.81, 47.87)	1869
Quintile 3	63.99 (60.98, 66.89)	1820
Quintile 4	81.52 (77.82, 84.72)	1707
Quintile 5 (richest)	88.18 (85.23, 90.61)	1422
**Education status**		
No education	37.92 (34.94, 41.00)	2278
Primary school	59.57 (56.59, 62.49)	3219
Secondary school +	81.34 (79.00, 83.47)	2995
**Place of residence**		
Rural	39.41 (36.37, 42.53)	3046
Urban	73.76 (71.34, 76.04)	5448
**Subnational region**		
Cabinda	63.84 (55.47, 71.45)	191
Zaire	79.64 (74.09, 84.26)	186
Uige	38.06 (30.22, 46.58)	460
Luanda	83.23 (79.43, 86.44)	2696
Cuanza norte	53.00 (43.69, 62.11)	111
Cuanza sul	31.52 (24.64, 39.31)	676
Malanje	53.16 (46.56, 59.65)	323
Lunda norte	36.94 (29.65, 44.88)	247
Benguela	58.05 (51.92, 63.94)	754
Huambo	65.41 (59.88, 70.54)	650
Bie	49.15 (42.16, 56.18)	414
Moxico	36.65 (29.80, 44.08)	167
Cuando Cubango	41.40 (33.99, 49.22)	164
Namibe	67.77 (58.95, 75.48)	108
Huila	48.24 (41.68, 54.87)	763
Cunene	61.06 (55.30, 66.52)	321
Lunda sul	60.11 (54.37, 65.59)	163
Bengo	64.78 (54.64, 73.73)	92
**National average**	61.4464	8492

**Fig. 1:**
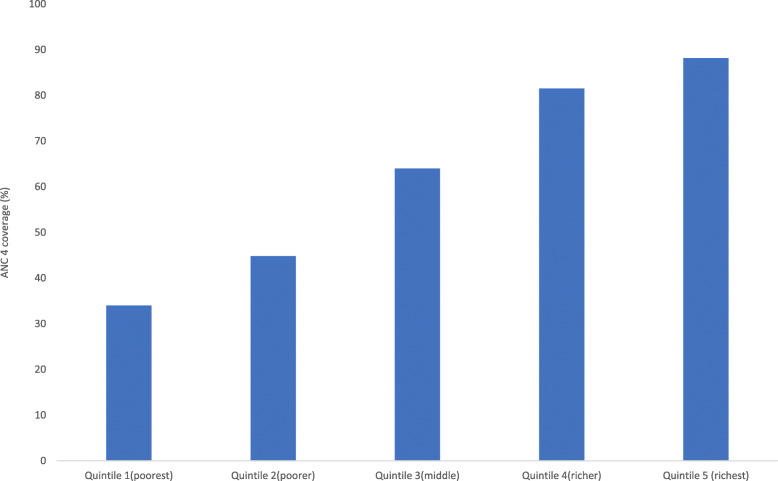
Coverage of ANC services across wealth quintiles in Angola: Evidence from 2015 Angola demographic and health survey

Coverage varied considerably across wealth quintiles. For instance, in the richest subpopulation, ANC coverage was greater as compared to the rich and middle quintile. Similarly, compared to the poorest quintile, the middle and poorer populations had better coverage (Fig. 1).

**Fig. 2:**
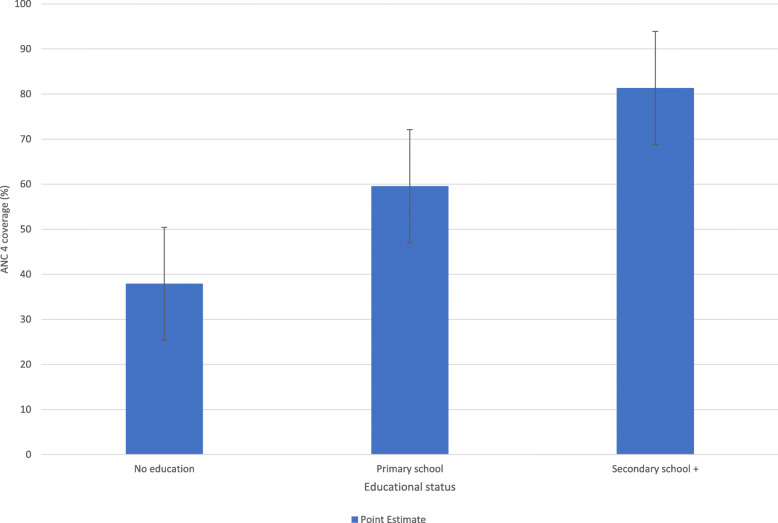
ANC service coverage based on maternal educational level in Angola: Evidence from 2015 Angola demographic and health survey

Figure 2 illustrates better coverage among subpopulations with secondary school education or higher compared to those with no formal education and primary school education.

**Fig. 3:**
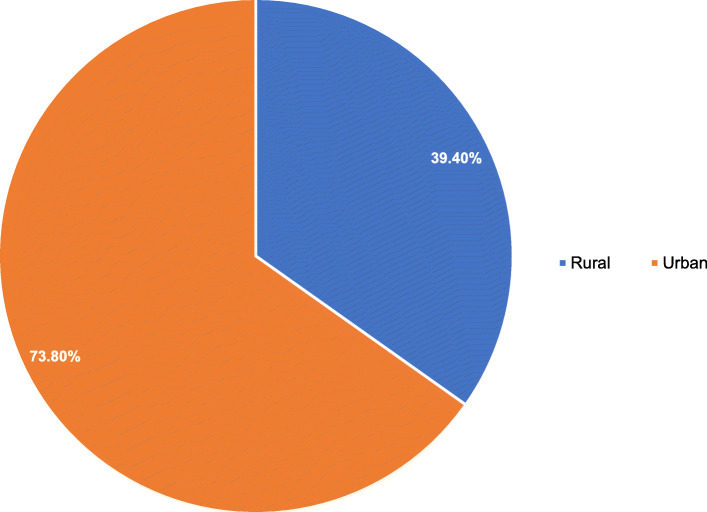
ANC coverage among urban-rural subpopulation in Angola: Evidence from 2015 Angola demographic and health survey

The results also show a difference in ANC coverage across the urban-rural subpopulation. In 2015, urban residents in Angola utilized ANC services more than rural residents (Fig. 3).

**Fig. 4:**
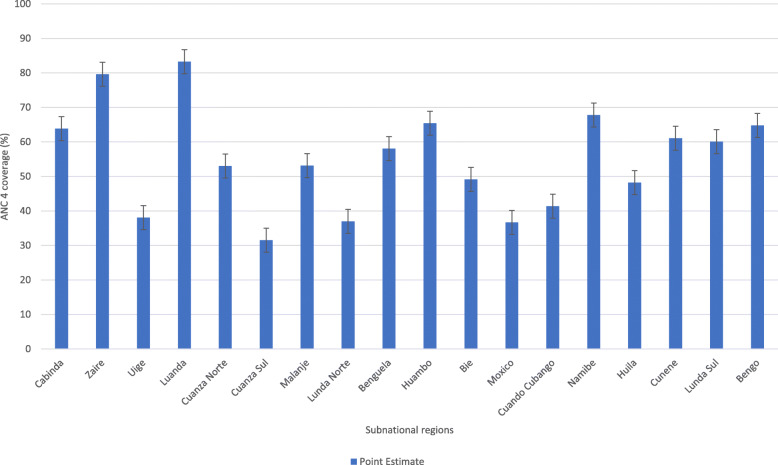
Coverage of ANC services across subnational regions in Angola: Evidence from 2015 Angola demographic and health survey

There was variation in subnational regions a well. In Luanda and Zaire regions, greater ANC coverage was observed whereas, other regions such as Cuanza Sul, Mexico and Lunda Norte had lower utilization of ANC (Fig. 4).

### Magnitude of inequalities

Table 2 shows profound socio-economic, regional and urban-rural disparities in ANC service utilization in 2015 favouring advantaged subpopulations. More specifically, we looked at substantial absolute and relative wealth-driven disparities in ANC service utilization both by simple (D, R) and complex (PAR, PAF) measures in 2015. The Difference measure 54.2% (95% UI; 49.59, 58.70) and PAF measure 43.5% (95% UI; 40.12, 46.92) indicated significant absolute and relative disparities respectively, favouring advantaged subpopulation such as richest and rich as compared to poorest and poor subpopulations. It means ANC coverage among the richest women was higher by 54 percentage points (pp) as compared to the poorest. Additionally, the utilization among the richest women was 2.6 times higher than the poorest. If the country had avoided both the absolute and relative wealth-related disparities, the 2015 national ANC coverage could be increased by an estimated 26.7 pp. (PAR), and 43.5 pp. (PAF), respectively.

**Table 2:** Regional variation and socio-economic inequalities in ANC coverage: Evidence from 2015 Angola demographic and health survey

Dimensions of inequality	Summary measures	Estimate [95% UI]
Economic status	D	54.15 (49.59, 58.70)
	PAF	43.52 (40.12, 46.92)
	PAR	26.74 (24.65, 28.83)
	R	2.59 (2.29, 2.88)
Education	D	43.41 (39.65, 47.17)
	PAF	32.38 (29.53, 35.23)
	PAR	19.89 (18.14, 21.64)
	R	2.14 (1.96, 2.32)
Place of residence	D	34.35 (30.47, 38.22)
	PAF	20.04 (17.70, 22.38)
	PAR	12.31 (10.88, 13.75)
	R	1.87 (1.71, 2.02)
Subnational region	D	51.70 (43.56, 59.85)
	PAF	35.45 (29.91, 40.99)
	PAR	21.78 (18.38, 25.18)
	R	2.64 (2.01, 3.26)

Broad absolute and relative education-related inequalities in ANC coverage were observed using all measures (D, PAR, PAF, R) not favouring the disadvantaged subpopulation. The Ratio measure of 2.1 (95% UI; 1.96, 2.32) and the PAR measure of 19.9 (95% UI; 18.14, 21.64) indicate significant relative and absolute disparities with disproportionately lower ANC coverage among non-educated and primary school educated subgroups compared to the secondary school or higher educated subgroup. For instance, we found a disproportionately 43.4 pp. excess ANC coverage among women who had had secondary school and above levels of education as compared to women who had no formal education. The findings also showed that the 2015 ANC national coverage could have been improved by an estimated 32.4 pp. (PAF) or 19.9 pp. (PAR) if the country had no education-related inequalities among the subgroups.

Pro-urban disparities in ANC service utilization were also observed. The PAF measure of 20% (95% UI; 17.70, 22.38) and the PAR measure of 12.3% (95% UI; 10.88, 13.75), revealed significant absolute and relative urban-rural disparities in ANC service utilization, favouring the urban residents. Pregnant women who lived in urban settings utilized ANC services 1.8 times (95% UI; 1.71, 2.02) more than rural residents. If the country evaded absolute and relative urban-rural disparities, the 2015 ANC coverage could be improved by 12.3 pp. (PAR) and 20.04 pp. (PAF).

We noticed significant subnational region inequalities in ANC service utilization both by simple (D, R) and complex (PAF, PAR) measures favouring subpopulation in regions like Luanda and Zaire. For instance, coverage among women living in the Luanda region was 51.7 pp. higher than in Cuanza Sul region. More specifically, the utilization in Luanda region was 2.6 times higher as compared to Cuanza Sul region. The 2015 ANC coverage could be increased by 35.5 pp. and 21.8 pp. if the country had cut the relative (PAF) and absolute (PAR) regional variation, respectively (Table 2).

## Discussion

Roughly 810 maternal deaths occur every day as a result of preventable pregnancy and childbirth-related causes, and 94% of these deaths occur in low-and lower-middle-income countries [34] . Even though ANC service plays a crucial role in averting the preventable maternal mortality, socio-economic inequalities still hinder many mothers from using the service [15–19].

To the best of our knowledge, this is the first study to assess inequalities in the use of ANC services in Angola using standardized methods to stratify the health inequalities. We found socio-economic, urban-rural and regional disparities with greater use of ANC services among pregnant women who were rich, educated, residing in urban settings and in regions like Luanda. These findings have provided an elaborate understanding of the coverage and magnitude of socio-economic, urban-rural and regional inequalities in the use of ANC in Angola. The novelty of these findings lies in their contribution to the contextual understanding of disparities in access to maternal healthcare in the midst of governmental policies to improve healthcare access.

Consistent with available literature [19, 35], we noticed extensive pro-rich disparities in the utilization of ANC services in Angola. The logical explanation for better ANC uptake among rich/richest women could be that women in the lower wealth quintiles (poorer or poorest) may not be able to afford the medical and non-medical costs associated with using ANC [36, 37]. Financial challenges may prevent poor women from attending ANC at all, limit the number of ANC visits or prolong the timing of ANC. The effects of socioeconomic status on the use of ANC services have been documented in other studies [37, 38]. Despite the presence of free (or subsidized) maternal health services for women in some African countries, women still bear some direct out of pocket medical costs (i.e. laboratory testing) and non-medical costs (i.e. transportation), posing financial barriers to the use of ANC services [39, 40]. Additionally, utilization, early timing of ANC visits and attendance at the recommended number of ANC visits are reduced in women who do not have health insurance [41]. Women’s desire to use ANC services during pregnancy is also hindered by their inability to pay for the services due to their low-income status [41, 42].

Similar to previous studies, our findings have shown a higher utilization of ANC services by educated women compared to women with no formal education in Angola [43, 44] Evidence suggest that health knowledge remains the important factor that explains the observed association between higher level of maternal education and use of maternal health care services including ANC [45]. The evidence further asserts that as a woman acquires more accurate information about a wide range of information on different health topics, she is more likely to use maternal health services. We have seen better ANC services usage among urban residents in Angola. Other studies have also identified the effect of urban residence on the use of ANC services [46, 47]. This might be explained by women in rural settings having to traverse long distances before receiving maternal health services [48]. Further, lack of transport, user fees, poorly staffed and ill-equipped institutions with poorly skilled personnel are problematic for women living in rural areas [48].

In our study, we found significant regional variation in ANC uptake across several regions in Angola. Our findings are comparable to the available evidence [43]. The reasonable explanation is the difference in remoteness, road and transport access, accessibility of health facilities, skilled health personnel and quality of care in the health care facilities [49].

These findings have provided an understanding of the need to ensure the effective implementation of the Angola National Health strategy 2016 which seeks to guarantee the provision of an essential health care package, mobilize communities, strengthen partnerships and promote health to ensure access to primary health care for the entire population by strengthening the municipal health system [50]. These socio-economic, rural-urban and subnational level disparities in access to ANC contradict the vision of Angola’s National Health Development Plan 2012–2025 which aims, among other things, to reduce maternal and child mortality through access to healthcare for all mothers [50]. Therefore, there is a need for the implementers of this Health Development Plan to revisit the aims, objectives and activities guiding the Health Development Plan to ensure that it considers the needs of disadvantaged groups in terms of healthcare access.

### Strengths and limitations

The study has a few strengths. First, inequality in ANC service utilization was examined using the WHO recommended Health Equity Assessment toolkit, which comprises various summary measures of the same phenomenon to assess inequality of different dimensions. This method has the potential to provide policymakers with multiple perspectives; consequently, it avoids the implementation of evidence-based strategies generated by a single or two summary measure that looks at average national-level data. Secondly, the study used the established WHO Health Equity Monitor Database (HEMD), which houses data from the Demographic Health Surveys, Multiple Indicator Cluster Surveys, and Reproductive Health Surveys from 112 countries. Using this HEMD allows comparable results to other published work based on the same source. There are also limitations in the study. We do not make any attempt to delve into the underlying causes of the observed ANC utilization disparities; we suggest future studies to explore factors that could explain the inequalities in the use of ANC services across different equity stratifiers. This can be done by utilizing different statistical approaches which would create models (i.e. regression analysis, decomposition analysis) to explore factors (i.e. distance to health facility & insurance) that explain the inequalities in the use of ANC services across equity stratifiers in Angola.

## Conclusion

Disparities in the use of ANC services have been to the detriment of poor and uneducated women, as well as women residing in rural areas or specific regions of Angola. Our findings have also shown the extent of the current disparity status using a variety of summary measures. We firmly recommend the need to improve upon the implementation of maternal healthcare policies that function best in the context of disadvantaged sub-populations to improve the utilization of ANC services. Such policies should include interventions aimed at strengthening media coverage to motivate mothers to access ANC services regardless of their wealth status, level of education or place of residence. To meet the needs of socio-economically disadvantaged women, community-based information centres can also be used as modes of providing education on ANC. Additionally, efforts should focus on training and motivating community health volunteers to provide home visits, counselling and identify mothers who require special ANC care. This can be enhanced through referrals to the next level health facility. All these policy interventions can help meet the 2030 Sustainable Development Goal (SDG) and reduce maternal mortality ratio (MMR) to 70/100, 000 live births. Further studies are essential to investigate underlying layers of inequities that lead to ANC inequality; especially for developing nations like Angola to be able to overcome the problems at a low cost.

## Data Availability

The datasets generated and/or analyzed during the current study are available in the WHO’s HEAT version 3.1 [https://www.who.int/gho/health_equity/assessment_toolkit/en/].

## Abbreviations

ANC: Antenatal Care
UI: Uncertainty Interval
D: Difference
DHS: Demographic and Health Survey
EA: Enumeration Area
EDHS: Ethiopia Demographic and Health Survey
HEAT: Health Equity Assessment Toolkit
ICF: Inner City Fund
PAR: Population Attributable Risk
PAF: Population Attributable Fraction
PCA: Principal Component Analysis
PPS: Probability Proportional to Size
R: Ratio
SDG: Sustainable Development Goal
WHO: World Health Organization
